# Removal of Brilliant Green Dye from Water Using *Ficus benghalensis* Tree Leaves as an Efficient Biosorbent

**DOI:** 10.3390/ma16020521

**Published:** 2023-01-05

**Authors:** Salma Gul, Azra Gul, Hajera Gul, Rozina Khattak, Muhammad Ismail, Sana Ullah Khan, Muhammad Sufaid Khan, Hani Amir Aouissi, Andrejs Krauklis

**Affiliations:** 1 Department of Chemistry, Women University Swabi, Swabi 22101, Pakistan; 2 Department of Chemistry, Shaheed Benazir Bhutto Women University, Peshawar 25000, Pakistan; 3 Department of Chemistry, University of Malakand, Chakdara 18800, Pakistan; 4 Scientific and Technical Research Center on Arid Regions (CRSTRA), Biskra 07000, Algeria; 5 Laboratoire de Recherche et d’Etude en Aménagement et Urbanisme (LREAU), Université des Sciences et de la Technologie (USTHB), Algiers 16000, Algeria; 6 Environmental Research Center (CRE), Badji-Mokhtar Annaba University, Annaba 23000, Algeria; 7 Institute for Mechanics of Materials, University of Latvia, Jelgavas Street 3, LV-1004 Riga, Latvia

**Keywords:** biosorbent, adsorption, water remediation, *Ficus benghalensis*, brilliant green, dye removal, natural, modeling, Freundlich, kinetics

## Abstract

The presence of dyes in water stream is a major environmental problem that affects aquatic and human life negatively. Therefore, it is essential to remove dye from wastewater before its discharge into the water bodies. In this study, Banyan (*Ficus benghalensis, F. benghalensis*) tree leaves, a low-cost biosorbent, were used to remove brilliant green (BG), a cationic dye, from an aqueous solution. Batch model experiments were carried out by varying operational parameters, such as initial concentration of dye solution, contact time, adsorbent dose, and pH of the solution, to obtain optimum conditions for removing BG dye. Under optimum conditions, maximum percent removal of 97.3% and adsorption capacity (Qe) value of 19.5 mg/g were achieved (at pH 8, adsorbent dose 0.05 g, dye concentration 50 ppm, and 60 min contact time). The Langmuir and Freundlich adsorption isotherms were applied to the experimental data. The linear fit value, R^2^ of Freundlich adsorption isotherm, was 0.93, indicating its best fit to our experimental data. A kinetic study was also carried out by implementing the pseudo-first-order and pseudo-second-order kinetic models. The adsorption of BG on the selected biosorbent follows pseudo-second-order kinetics (R^2^ = 0.99), indicating that transfer of internal and external mass co-occurs. This study surfaces the excellent adsorption capacity of Banyan tree leaves to remove cationic BG dye from aqueous solutions, including tap water, river water, and filtered river water. Therefore, the selected biosorbent is a cost-effective and easily accessible approach for removing toxic dyes from industrial effluents and wastewater.

## 1. Introduction

Water is the most crucial necessity for the existence of life on earth. It is not possible to sustain life on earth without water. Water resources include oceans, rivers, lakes, canals, and underground water. Due to ever-increasing ecological pollution, these resources are being polluted with poisonous materials [1,2] because of untreated discharges from local sewage, pharmaceutical by-products, and industrial effluents. Industrial effluents contain dyes because 10–15% are released during the dying process. Approximately 10,000 different types of dyes, approximately 7 × 10^5^ tons, are produced annually worldwide. It is widely used in many industries, such as textiles, foods, cosmetics, pharmaceuticals, photography, plastics, and paper [3,4]. The textile industry releases the highest amount of dye effluent, namely 54% of the dyes, contributing to more than half of the existing dye effluents seen in the environment around the world. Some other industries are also known to produce high amounts of dye effluents from various associated processes. The dyeing industry produces 21%, the paper and pulp industry produces 10%, the tannery and paint industry produces 8%, and the dye manufacturing industry produces 7% in their effluent [5].

When discharged untreated, these dyes pose a severe environmental risk because pigments in water absorb and reflect sunlight. Hence, these dyes reduce the photosynthetic activity of aquatic plants [6], such as algae, and affect the food chain. Many dyes and their breakdown products cause cancer, mutations, and toxicity [7]. It also affects human beings. For instance, due to these dyes, the kidneys, liver, and the nervous system fail their function [8]. Permanent hair dyes contain a component called paraphenylenediamine (PPD), commonly known as an allergen that causes allergic reactions. This dye also causes redness and swelling in sensitive areas. Dyes containing persulfates and prolonged inhalation can cause coughing, cancer, lung inflammation, and asthma [9].

One of the most commonly used colors in the paper printing and textile industries is the brilliant green (BG) dye (Figure S1) [10]. It is used to dye materials, particularly silk and wood, and is also present in plastic and rubber industries’ effluents. Skin contact, eye contact, and the ingestion of this dye are all dangerous. It is harmful to the lungs when inhaled. The material can harm target organs when exposed repeatedly or for an extended period. Its breakdown produces nitrogen oxides, carbon dioxide, and sulphur oxides. Therefore, getting the BG dye out of the water is crucial. Due to its toxic nature, many researchers are working to remove BG from wastewater [11,12,13,14,15,16,17,18].

Various treatment technologies are used to remove dyes from wastewater, such as chemical coagulation-flocculation, oxidation processes, biological processes, membrane-based separation processes, bioremediation [19], and adsorption [20,21,22,23]. Each of the aforementioned procedures has its advantages and disadvantages. Among them, adsorption on solid surfaces is gaining popularity due to its easy maintenance, low-cost operation, and large-scale application [24]. A common adsorbent is activated carbon because of its high surface area and adsorption capacity [25]. However, the technique is not profitable for industrial applications due to its high cost.

Therefore, many researchers have investigated the feasibility of low-cost adsorbent materials, such as banana pith, waste orange peel, de-oiled soya, bottom ash, and rice husk [26]. The BG dye was removed from water in this work using *Ficus benghalensis* (*F. benghalensis*) tree leaves as an adsorbent. BG dye could be effectively removed from water using *F. benghalensis* tree leaves without the need for any prior adsorbent treatment, which is a cost-effective method of treating water, and or, wastewater. The best results of the adsorption process may be achieved in a short amount of time, at a reasonable cost, after the experimental circumstances are tuned. The adsorption process is influenced by a number of reaction parameters, including temperature, pH, ionic strength, adsorbent dose, and adsorbate characteristics [27,28]. Optimizing reaction conditions is critical as a result. The reaction conditions in this study were optimized for the elimination of BG in aqueous medium. Additionally, the effectiveness of the adsorbent was evaluated in tap water, river water, and filtered river water, with BG dye exhibiting good adsorption efficiency. Thus, *F. benghalensis* tree leaves can be used commercially as an adsorbent without undergoing any prior chemical processing, acting as a successful adsorbent for the removal of hazardous dyes from actual wastewater.

## 2. Materials and Methods

### 2.1. Materials

In this study, *F. benghalensis* tree leaves were used as the adsorbent, while adsorbate was cationic dye, i.e., brilliant green (BG), C_27_H_34_N_2_O_4_S. 0.5 M HCl (hydrochloric acid, Merck 37%, Rahway, NJ, USA) and NaOH (sodium hydroxide, pellet, Sigma Aldrich ≥ 98%, St. Louis, MO, USA) solutions, were also used to maintain the pH of solutions. Distilled water was used for solution preparation and ethanol (Merck, Darmstadt, Germany) for rinsing the glassware.

### 2.2. Equipment

For proper mixing of the biosorbent and dye, a digital orbital shaker was used at 200 rpm at room temperature (a product of PCSIR Pakistan), whereas a UV-Visible spectrophotometer was used to measure the absorbance (model UV 3000, Analytik/Jene, Hamburg, Germany).

### 2.3. Preparation of Adsorbent

Banyan tree leaves were collected from Swabi, Khyber Pakhtoonkhwa (KPK) province of Pakistan. After the collection of the plant leaves, first, all the leaves were carefully removed. Then, they were washed several times with tap water and then one to two times with distilled water to remove all dust particles. After that, the leaves were dried in sunlight for several days and then powdered using mortar. This powdered form of leaves was then used for the adsorption experiment. A similar procedure was carried out for the preparation of adsorbent from the bark and the performance of both leaves and bark was compared.

### 2.4. Preparation of Dye Solution

The stock solution (500 mg/L) of BG was prepared by dissolving 0.5 g of dye in 1000 mL of distilled water. Then, the stock solution was diluted to prepare experimental solutions of the desired concentration.

### 2.5. Determination of the Adsorption Efficiency of F. benghalensis Tree Parts

All of the experiments were carried out at 625 nm since, according to the literature, BG dye exhibits maximal adsorption at this wavelength [29]. A comparison of the leaves and bark of Banyan (*F. benghalensis*) tree sections for the adsorption of cationic BG dye was done through experiments. For this, 50 mg of dye was dissolved in 1 L of distilled water to create a 50-ppm solution of BG. A pipette was then used to transfer 20 mL of the produced solution into two 100 mL beakers. The UV/Vis spectrophotometer was used to measure their absorption. Each beaker holding 20 mL of BG solution received 50 mg of *F. benghalensis* leaves or bark before being placed on a digital orbital shaker for approximately one hour. Following this, filter paper was used to filter both solutions, and their absorbance was measured. Finally, the final dye solution concentration was determined using the Beer–Lambert law [30,31], and it is as follows:

(1) A = εcl 

where A = absorbance of the solution, ε = molar absorptivity of the dye, c = concentration of the solution, and l = path length of the cuvette (i.e., 1 cm). 

The value of Qe (mg/g), which represents the amount of dye adsorbed on the surface of the adsorbent, was calculated using the formula given below [32]:

(2) $$\mathrm{Qe}=\frac{\left(\mathrm{Ci}-\mathrm{Ce}\right)}{m}V\;$$

where Ci (ppm) is the initial concentration of dye solution before adsorption, Ce (ppm) is the equilibrium concentration of dye solution, V (L) is the volume of solution, and m (g) represents the mass of the adsorbent.

Similarly, % removal was calculated using the following formula [32]:

(3) $$\%\;\mathrm{Removal}=\frac{\mathrm{Ci}-\mathrm{Ce}\;}{\mathrm{Ci}}\times 100$$

## 3. Results and Discussion

### 3.1. Comparison of Adsorption Performance of Different Sections of F. benghalensis Tree for BG Dye Removal

**Figure 1:**
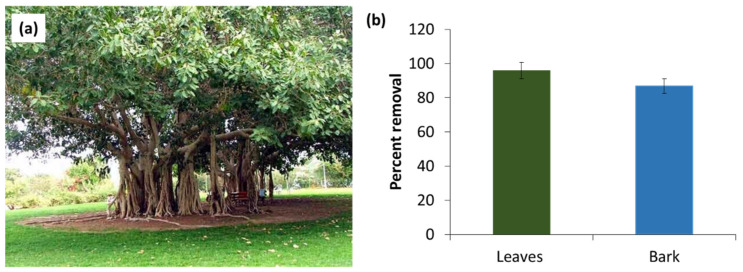
(**a**) *F. benghalensis* tree. (**b**) Comparison of adsorption performance of *F. benghalensis* tree leaves and bark to remove BG dye.

Different parts of the *F. benghalensis* tree, i.e., leaves and bark, were taken as adsorbents, and their efficiency for BG percent removal were determined (Figure 1). Results showed that the percent removal value of leaves was greater than bark at 625 nm. Therefore, the leaves were chosen as the adsorbent for further study to remove BG dye.

### 3.2. Effect of Contact Time on the Process of Adsorption

**Figure 2:**
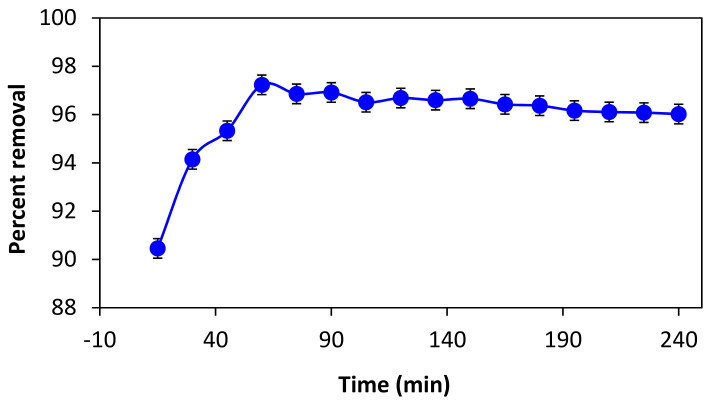
Effect of contact time on percent removal of dye.

Shaking and the amount of time the adsorbent is in contact with the adsorbate before equilibrium between adsorption and desorption is reached have an impact on the adsorption process. Adsorption is a surface phenomenon. Thus, shaking will significantly affect how quickly it accelerates to equilibrium. A 50-ppm stock solution of the dye was prepared in order to optimize the shaking and contact time for BG dye removal. It was then divided using a pipette into 15 solutions, each with a volume of 20 mL, and placed in 100 mL beakers. A UV/Vis spectrophotometer was used to record their absorbance. All solutions received 50 mg of adsorbent (leaves powder) before being put on a digital orbital shaker. At 200 rpm, each solution was shaken for various contact times. The first solution, for instance, was shaken for 15 min, the second for 30 min, the third for 45 min, and so on up to 300 min. The final absorbance values of these solutions were recorded after they had been filtered with filter paper. Figure 2 makes it evident that the percent removal reached its highest at 60 min and then gradually fell as time went on. Therefore, it was observed that 60 min was the ideal amount of time to reach equilibrium for the adsorption of BG dye using *F. benghalensis* leaves. Shaking and more adsorbent–adsorbate interaction time tend to change the equilibrium toward desorption, which decreased the percentage of BG removed.

### 3.3. Effect of Adsorbent Dose (Amount of Leaves Powder) on the Adsorption of BG

**Figure 3:**
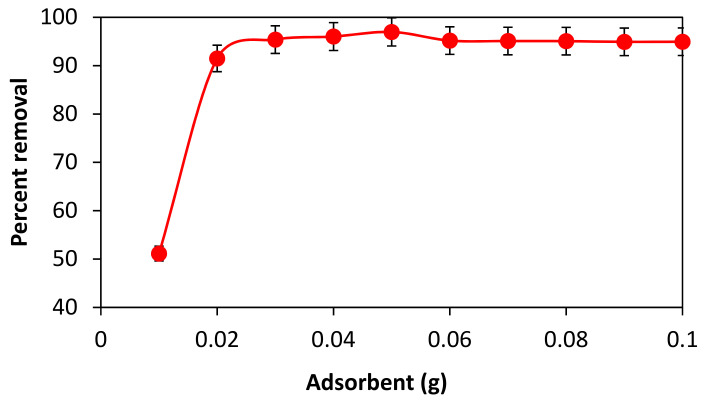
Effect of adsorbent dose on percent removal of BG dye.

The adsorption process is influenced by the amount of the adsorbent. To determine the ideal adsorbent dose, a 50-ppm solution of BG dye was made, distributed into ten beakers (20 mL each), and their absorbance was measured. Then, various doses of adsorbent (i.e., 10 to 100 mg) were added to each dye solution. For 60 min, all of the solutions were shaken at 200 rpm. Filtration was then performedto keep track of the absorbance. Figure 3 shows that the percentage of dye removal rose as adsorbent dosage was increased up to a certain point before becoming constant.

The accessible surface area and active sites for the interaction between the powdered leaves and BG dye increase with an increase in adsorbent dosage (quantity of powdered leaves), resulting in increased dye adsorption [33]. The surface loading of adsorbent by adsorbate reaches its maximum at low adsorbent doses without further availability of the active sites, and the leftover BG continues to be a component of the aqueous solution. Since there are more sites for dye loading as the adsorbent dosage rises, the amount of BG removed also rises. The BG dye removal efficiency reaches its peak at 50 mg of adsorbent for 50 ppm BG, and from that point on, adding more adsorbent has no further impact on the adsorption process, even though constant percent removal rates continue to apply.

### 3.4. Effect of pH on the Adsorption of Brilliant Green

**Figure 4:**
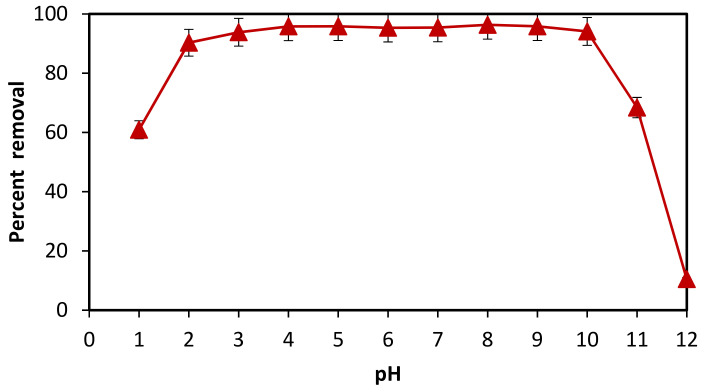
Effect of pH on percent removal of BG dye by *F. benghalensis* leaves powder.

The pH of dye solution at which the most adsorption takes place was determined by an adsorption investigation at various pH levels. Twelve (12) 100 mL beakers with 20 mL of BG each were filled with a 50 ppm solution of BG. 0.5 M HCl and/or NaOH solutions were used to change the pH of these solutions from 1 to 12. The optimal adsorbent dose (50 mg) was then added to all solutions, and all solutions were then shaken for 60 min at 200 rpm as before. Every solution’s absorbance was measured both before and after adsorption. Figure 4 illustrates how the adsorbent displayed varying adsorption efficiencies for the same dye at various pH levels.

This might be caused by a change in the adsorbent’s surface charge, the adsorbate’s degree of ionization, or the extent to which functional groups on the adsorbent’s active sites have been dissociated [34]. The pH controls different charges on the adsorbent surface and regulates the ionization of dye molecules. It is implied that pH affects the adsorption process. The dye’s altered structure causes it to change color when the pH changes. The adsorption efficiency improves from 1 to 8 as the pH value rises. High adsorption took place at pH 8, after which the adsorption value fell [35]. Consequently, pH 8 is the optimum pH for removing BG dye and was used for further study.

### 3.5. Effect of Initial BG Dye Concentration on Its Adsorption

**Figure 5:**
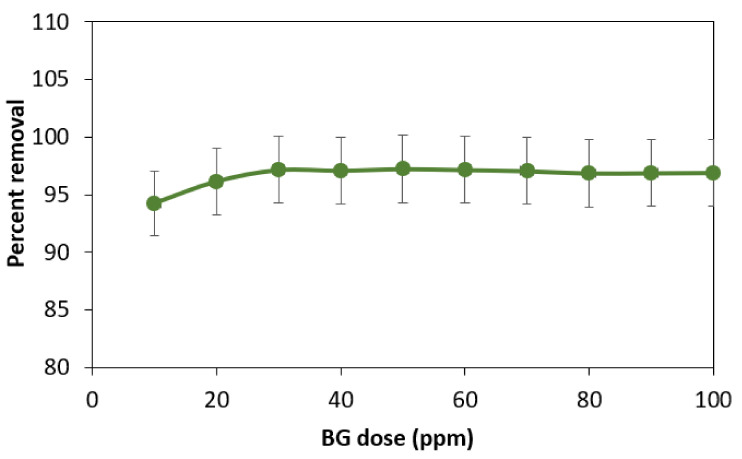
Effect of initial dye concentration on the percent removal of BG by selected adsorbate.

A 200-ppm stock solution of BG in distilled water was prepared to examine the effect of the BG’s initial concentration on its adsorption. In the concentration range of 10–100 ppm, several BG diluted solutions were prepared (20 mL each). Every solution was added a 50 mg adsorbent, and then it was shaken as usual for up to an hour. Following filtration after adsorption, according to a prescribed process, the absorbance of each solution was measured before and after adsorption. Equations (1)–(3) were used to compute the adsorbent’s capacity (Qe) and percent removal of BG. According to Figure 5, the adsorption effectiveness of *F. benghalensis* leaves improves with an increase in the dose of the adsorbate (BG dye).

The percent removal value climbed to 97.26 when dye concentration increased from 10 to 50 ppm, and similarly, the Qe value increased from 3.77 to 19.45. Equilibrium was reached at 50 ppm, and no more adsorption growth was seen after that (Figure 5). Because of more successful collisions as the initial dye concentration rises, the amount of dye removed rises up until the saturation of the active binding sites on the adsorbent surface. When the concentration of BG is increased further, the percentage of BG removal remains constant because there are no more binding sites that become available and because adsorption and desorption are in equilibrium. As a result, it is determined that 50 ppm is the optimum concentration at which 50 mg of Banyan tree leaves (an adsorbent) can adsorb the most. Above this concentration, no increase in adsorption was seen. This is due to the fact that more active sites on the surface of the adsorbent are available for dye adsorption at low doses. Dye molecules quickly adsorb on an adsorbent’s open binding sites. Repulsive forces between free and adsorbed dye molecules develop over time as the adsorbent surface becomes saturated, leading to low percent removal at high concentrations.

### 3.6. Comparison of Adsorption Efficiency of Ficus benghalensis Leaves Powder as an Adsorbent with Animal Charcoal and Silica Gel for BG Removal

**Figure 6:**
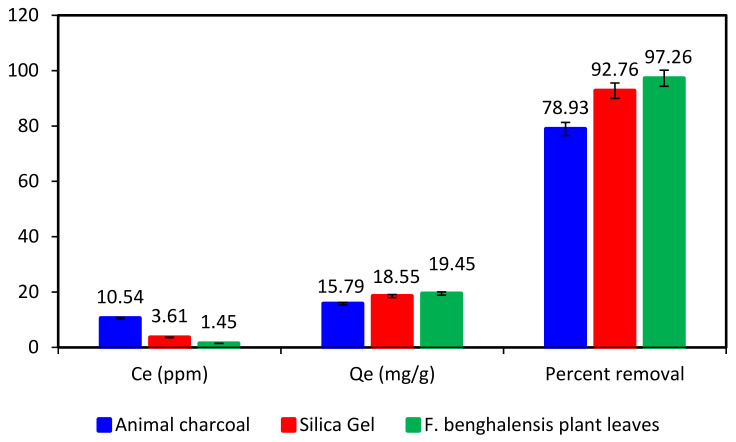
Comparison of equilibrium concentration (Ce), adsorption capacity (Qe), and percent removal efficiency of animal charcoal, silica gel, and *F. benghalensis* leaves powder for BG dye (adsorbate).

For the removal of BG, the effectiveness of several adsorbents was compared to our chosen biosorbent. This was accomplished by preparing a 50-ppm solution of BG dye and setting the pH to 8. Three 100 mL beakers each received a 20 mL solution, to which 50 mg each of animal charcoal, silica gel, and *F. benghalensis* leaves powder were added. The three beakers were then shaken on an orbital digital shaker for 60 min. Following filtration, the absorbance was measured both before and after adsorption. Figure 6 demonstrates that the powder from *F. benghalensis* leaves had a better affinity for the adsorption of BG dye than silica gel and animal charcoal, and when our biosorbent was compared to published literature Table 1, it produced outstanding results. Animal charcoal and silica gel only removed 78.93% and 92.76% of the BG dye, respectively, whereas the powdered leaves of *F. benghalensis* removed 97.26%. The Qe value of the powdered leaves of *F. benghalensis* was found to be higher than that of silica gel and animal charcoal. Thus, *F. benghalensis* leaves powder is a cost-effective and accessible adsorbent with a better adsorption capacity than traditional adsorbents.

### 3.7. Determination of Adsorption Performance of F. benghalensis Leaves Powder in Water Collected from Different Sources

**Figure 7:**
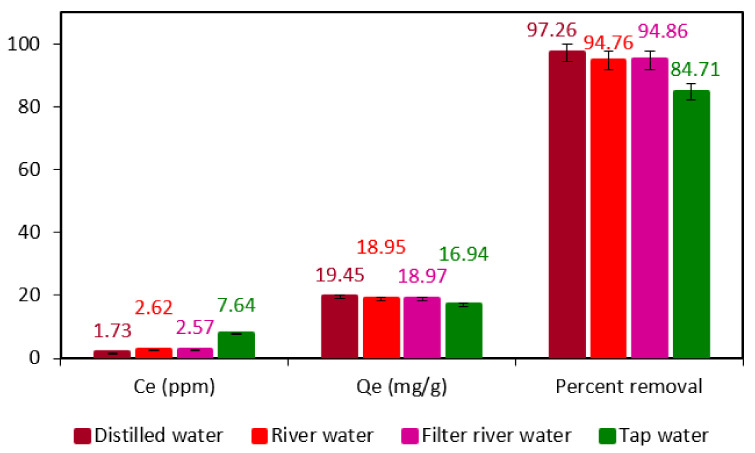
Adsorption performance of *F. benghalensis* leaves powder in various types of water and its corresponding equilibrium concentration (Ce), adsorption capacity (Qe), and percent removal efficiency for BG dye (adsorbate).

Water from several sources, including tap water, river water, filtered river water, and distilled water, were used in the experiment to determine the degree of adsorption. In order to achieve this, 50 ppm of BG dye was dissolved in a water sample obtained from various sources, the pH was maintained using 0.5 M HCl and 0.5 M NaOH, and the absorbance was measured. The optimum amount of adsorbent was then added to each of the solutions prepared in the various water samples, and they were shaken as usual for 60 min. Following this, filter paper was used to filter all of the solutions, and their absorbance was noted. In comparison to water gathered from other sources, the results indicated that the adsorption procedure carried out in distilled water had increased adsorption effectiveness. However, in every water sample, the adsorbent displayed excellent performance (Figure 7). As a result, this adsorbent can be used to effectively remove dyes from industrial wastewater contaminated by dyes.

### 3.8. Best-Fit Adsorption Isotherm for BG Removal by F. benghalensis Leaves Powder

The term “adsorption isotherm” describes the relationship between the concentration of the adsorbate in equilibrium and the amount of adsorbate adsorbed at a particular adsorbent at a constant temperature. The parameters acquired from the various models offer crucial details on the adsorption mechanisms as well as the adsorbent’s surface properties and affinities. The two models for single-solute systems that are most frequently used are the Langmuir and Freundlich adsorption isotherms [37,38]. As a result, the referred models were used for this study, which involved the BG-biosorbent system, and it was discovered that the data followed one of them. The interactions between the molecules or ions of the adsorbate and the adsorbent surface sites are best described by adsorption isotherms.

#### 3.8.1. Langmuir Isotherm Model

The Langmuir isotherm is based on homogeneous adsorption. The adsorbate cannot transmigrate in the surface plane and can only be absorbed at a fixed number of specific localized sites. The Langmuir model can be given as Equation (4).

(4) $$\frac{1}{Qe}=\frac{1}{K_{L}\cdot Qmax}\cdot \frac{1}{Ce}+\frac{1}{Qmax}\;$$

where Qe is the adsorption capacity (mg/g), Ce is the equilibrium concentration (mg/L), and K_L_ is the Langmuir isotherm constant.

#### 3.8.2. Freundlich Isotherm Model

The Freundlich isotherm was developed to describe the non-ideal, reversible, and multilayer adsorption [39]. It refers to the heterogeneous surface of the adsorbent. The linear form of the Freundlich isotherm model is represented by Equation (5).

(5) $$\mathrm{logQe}={\mathrm{logK}}_{F}+\frac{1}{n}{\mathrm{logC}}_{e}$$

where Qe is the adsorption capacity (mg/g), Ce is the equilibrium concentration (mg/L), K_F_ and 1/n are Freundlich isotherm constants related to adsorption capacity. A plot of log Qe vs. log Ce yields a straight line, with a slope and intercept equivalent to 1/n and log K_F_, respectively.

**Figure 8:**
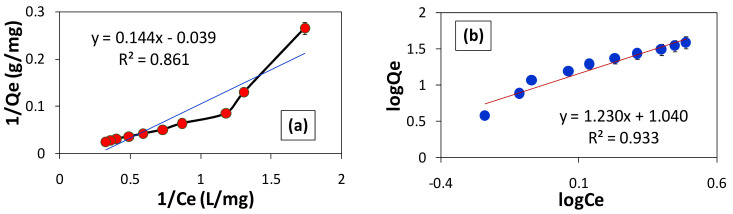
(**a**) Langmuir isotherm plot. (**b**) Freundlich isotherm plot.

The information obtained for the removal of BG by *F. benghalensis* leaves powder was applied to the linear Equations (4) and (5) of Langmuir and Freundlich adsorption isotherms (Figure 8). The outcomes demonstrate that, when compared to the Langmuir adsorption equation, the Freundlich adsorption equation provides the best linear fit for the data collected. Comparing the Freundlich linear plot’s R-squared value to the Langmuir linear plot’s R-squared value, the Freundlich linear plot has a linearity of 93%. Since our data fit the Freundlich adsorption isotherm rather than the Langmuir adsorption isotherm, the negative value of Qmax derived from the Langmuir linear fit equation serves as additional evidence (Figure 8a, Table 2). Since the adsorption capacity can never be negative, the adsorption data in this investigation follow the Freundlich adsorption isotherm. As explained by the Freundlich model, this assisted in identifying the non-ideal, reversible, multilayer, and heterogeneous adsorbent surface. Table 2 displays various parameters obtained from each plot.

### 3.9. Kinetics of BG Adsorption by F. benghalensis Leaves Powder 

To determine the order of adsorption, the time course graphs for the removal of BG by the powdered leaves of *F. benghalensis* were plotted. On the gathered data, the pseudo-order kinetic models were applied [40]. To evaluate the best linear fit and the order of the adsorption process for BG removal, the pseudo-first-order [41] and pseudo-second-order [42] linear equations were examined. The reaction pathway, dye adsorption mass transfer method, and diffusion rate are all vitally revealed by the process’ kinetics [43,44,45].

#### 3.9.1. Pseudo-First-Order (PFO) Kinetic Model

Equation (6) represents the linear equation of the pseudo-first-order kinetic model [35]. Here, qt and qe are the amounts of dye adsorbed (mg/g) at contact time t (min) and at equilibrium, respectively, and k_1_ is the pseudo-first-order rate constant (min^−1^).

(6) $$\mathrm{Log}\;\left(\mathrm{qe}\;–\;\mathrm{qt}\right)=\;\log\;\mathrm{qe}-\left(k_{1}/2.303\right)t\;$$

Equation (6) states that if an adsorption process exhibits first-order kinetics, a straight line with a slope equal to k_1_/2.303 and an intercept equal to log qe should be obtained when plotting log (qe – qt) against time (t). The intercept and slope of the plot can be used to determine the equilibrium adsorption capacity and the adsorption rate constant, k_1_.

#### 3.9.2. Pseudo-Second-Order (PSO) Kinetic Model

Equation (7) is the straight line equation of the pseudo-second-order kinetic model [46].

(7) $$\frac{t}{\mathrm{qt}}=\frac{1}{\left[k_{2}{\mathrm{qe}}^{2}\right]}+\frac{t}{\mathrm{qe}}$$

where k_2_ is the rate constant for the pseudo-second-order adsorption kinetics. If the process follows pseudo-second order, the plot of t/qt vs t should result in a straight line with a slope equal to 1/qe and an intercept equal to 1/k_2_qe.

**Figure 9:**
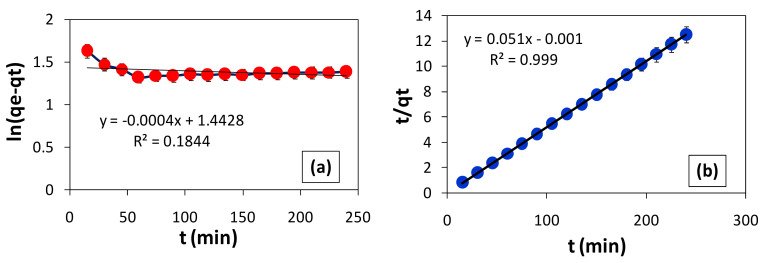
Kinetics of BG removal by *F. benghalensis* leaves powder. (**a**) Pseudo-1st-order; (**b**) pseudo-2nd-order plots.

The data collected in this work for BG removal by *F. benghalensis* leaves powder were used to implement the pseudo-first and pseudo-second order straight line equations. Because the data showed a better linear fit (R-squared value) for PSO than for PFO, the plots (Figure 9a,b) demonstrated that the adsorption process follows PSO rather than PFO. The kinetic parameters that are listed in Table 3 for comparison were calculated using the slope and intercept values of each plot (Figure 9a,b). The experimental value of qe, which is 19.45 mg/g, and the qe value, which was determined from Figure 9b, are nearly identical. The kinetic model (PSO), which identified the rate-determining phase involved in chemisorption, revealed the adsorption behavior by showing that the electrons are shared or transferred between *F. benghalensis* leaves powder surface active groups and cationic dye molecules [20].

### 3.10. Fourier Transform Infrared Spectroscopy of the Adsorbent before and after Adsorption

**Figure 10:**
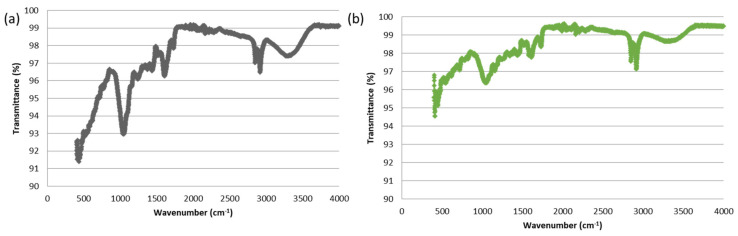
(**a**) FTIR spectrum of *F. benghalensis* leaves powder. (**b**) FTIR spectrum of *F. benghalensis* leaves powder after adsorption of brilliant green dye.

*Ficus benghalensis* leaves powder was characterized by FTIR spectroscopy. Figure 10a,b represent the FTIR spectra of the adsorbent (*F. benghalensis* leaves powder) before and after adsorption. The FTIR spectrum of *F. benghalensis* leaves powder showed different peaks at positions 432 cm^−1^, 447 cm^−1^, 1047 cm^−1^, 1606 cm^−1^, 2917 cm^−1^, 3291 cm^−1^, and 3389 cm^−1^. The strong and broad peak at 3389 cm^−1^ may correspond to O–H stretching vibration due to inter and intra-molecular hydrogen bonding of polymeric compounds, such as alcohol, phenols and a carboxylic acid, as in pectin, lignin and cellulose [47]. The peaks at 2917 cm^−1^ (strong band) and 3291 cm^−1^ (weak bands) represent the presence of symmetric and asymmetric CH stretching of aliphatic acid [48]. The medium revolution peak at 1606 cm^−1^ is because of C=O stretching of carboxylic acid with intermolecular hydrogen bonding. In other studies, this peak is described as the region of both ionized-non-coordinated and ionized coordinated COO− group [49]. A strong band at 1047 cm^−1^ indicates C-O-C stretching vibration [50]. As a result, the FTIR study indicates that *F. benghalensis* leaves powder contains functional group such as OH, COOH and CO, that are used as adsorption sites for the interaction of cationic brilliant green dye. The dye-loaded bioadsorbent’s FTIR spectrum revealed a noticeable difference in percent transmittance (intensity) at all peak points, indicating effective adsorption. These alterations in the adsorbent’s surface show that biosorption involved a potent sorption process, and the interaction between the dyes and the powdered leaves of the *F. benghalensis* tree was chemisorption. FTIR analysis thus supports the findings of the experiment.

### 3.11. Scanning Electron Microscopy (SEM) of the Adsorbent before and after Adsorption

**Figure 11:**
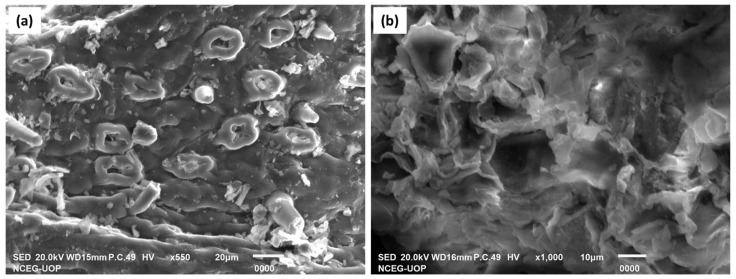
(**a**) SEM image of *F. benghalensis* leaves powder before adsorption. (**b**) SEM image of *F. benghalensis* leaves powder after adsorption of BG dye.

The surface morphology of the *F. benghalensis* leaves powder before dye adsorption is clearly seen in the SEM image (Figure 11a), with an uneven texture, a high surface roughness, and various levels of porosity, which offer potential sites for dye adsorption. The macropores and surface roughness of *F. benghalensis* leaves, which contribute to their high surface area and are necessary for the adsorption of a dye, make them good adsorbent. The SEM image (Figure 11b) revealed a smooth surface because the BG molecules are trapped on the surface of the adsorbent after dye has been adsorbed to the powdered leaves of *F. benghalensis*, covering the pores on the biosorbent.

### 3.12. Mechanism of Adsorption of Brilliant Green Dye on F. benghalensis Leaves

**Figure 12:**
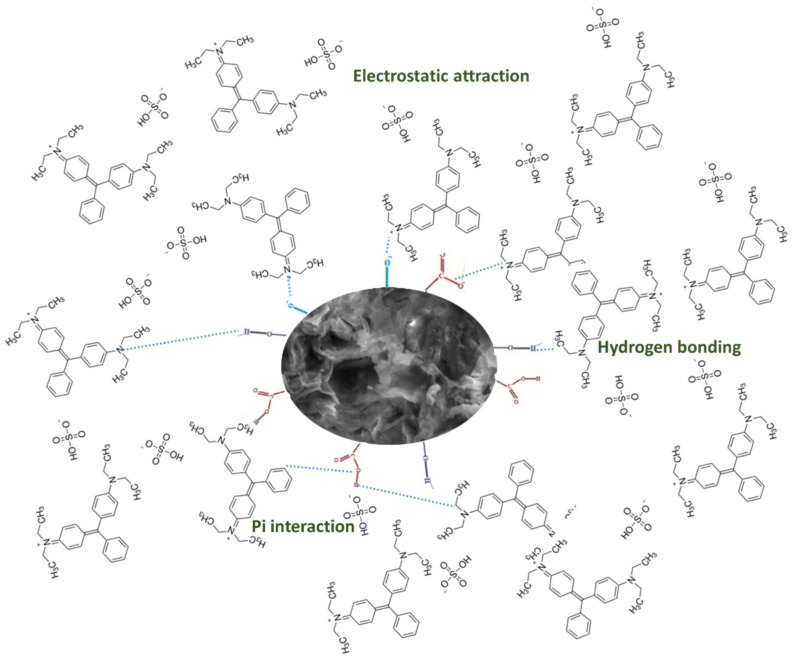
Mechanism of adsorption of BG dye on *F. benghalensis* leaves powder.

Figure 12 depicts the suggested BG dye adsorption mechanism on *F. benghalensis*. The functional groups that are present on the surface of the powdered *F. benghalensis* leaves are what cause the majority of the interactions, which include electrostatic attractions, hydrogen bonding, cationic exchange, and pi interaction. A negatively charged surface might be created by the functional group (-OH, -COOH), as seen by the FTIR spectrum in Figure 10. Positively charged dye molecules interact electrostatically with negatively charged *F. benghalensis* leaf powder, improving the adsorption process. The lone pair of electrons on the nitrogen atom in the cationic BG dye form a hydrogen bond with the H atoms in the functional group on the surface of the adsorbent. Cationic exchange also takes place between the absorbent and dye surfaces, and pi interaction is a significant contributor to BG adsorption on powdered *F. benghalensis* leaves. Pi interaction typically takes place when the oxygen atom’s lone pair electron is delocalized into the aromatic dye ring’s pi orbital.

## 4. Conclusions

The results of the current investigation indicate that *F. benghalensis* leaves are an effective adsorbent for the removal of BG dye from wastewater, with a maximum percent removal of 97.26 and a Qe value of 19.45 mg/g under optimum conditions. Additionally, the Freundlich isotherm is followed by the adsorption, showing heterogeneous adsorbent surfaces where multi-layer adsorption occurs. The experimental observations are consistent with the pseudo-second-order kinetic model, which suggests that chemisorption is the rate-limiting step. From this work, it may be inferred that Banyan tree leaves are effective at removing dyes from real wastewater, including river water and tap water in addition to distilled water. Thus, they can be used as an efficient adsorbent for real-world applications without any treatment required for conversion to the adsorbent.

## Figures and Tables

**Table 1:** Comparative analysis of different adsorbents for brilliant green removal.

Biosorbent	Initial Dye Conc. (ppm)	Adsorbent Dose (g)	Contact Time	Percent Removal	Reference
Red clay	20	0.4	4 h	96	[27]
Kaolin	20	1	90 min	91	[34]
EDTA-modified magnetic sawdust carbon nanocomposites	10	0.5	0.5 h	96.7	[36]
*Ficus benghalensis* tree leaves	50	0.05	60 min	97.3	Present work

**Table 2:** Parameters for the adsorption of BG by *F. benghalensis* leaves powder.

Langmuir Adsorption Isotherms				Freundlich Adsorption Isotherm		
Q_max_ (mg/g)	K_L_ (L/g)	R_L_	R^2^	K_F_ (mg/g)	1/n	R^2^
−25.64	−0.27	−0.066	0.86	10.98	1.23	0.93

**Table 3:** The kinetic parameter for the adsorption of BG dye on *F. benghalensis* leaves powder.

Pseudo-First-Order Kinetic Model			Pseudo-Second-Order Kinetic Model		
k_1_	q_e_	R^2^	k_2_	q_e_	R^2^
0.00092	27.72	0.18	−2.603	19.6	0.99

## Data Availability

Not applicable.

## Supplementary Materials

The following supporting information can be downloaded at: https://www.mdpi.com/article/10.3390/ma16020521/s1, Figure S1: Structural formula of brilliant green dye.
