# Supplementary material for: Removal of Brilliant Green Dye from Water Using Ficus benghalensis Tree Leaves as an Efficient Biosorbent
Source: Materials (Basel). 2023 Jan 5;16(2):521. doi: 10.3390/ma16020521 (PMC9866320; doi:10.3390/ma16020521)
Supplement: Supplementary file 1 [file materials-16-00521-s001.zip › materials-2016004-supplementary.pdf]

materials

Supplementary Information

Removal of Brilliant Green Dye from Water Using *Ficus benghalensis* Tree Leaves as an Efficient Biosorbent

Salma Gul [1,*], Azra Gul [1], Hajera Gul [2], Rozina Khattak [2,*], Muhammad Ismail [1], Sana Ullah Khan [1], Muhammad Sufaid Khan [3], Hani Amir Aouissi [4,5,6] and Andrejs Krauklis [7]

1 Department of Chemistry, Women University Swabi, Swabi 22101, Pakistan
2 Department of Chemistry, Shaheed Benazir Bhutto Women University; Peshawar 25000, Pakistan
3 Department of Chemistry, University of Malakand, Chakdara 18800, Pakistan
4 Scientific and Technical Research Center on Arid Regions (CRSTRA), Biskra 07000, Algeria
5 Laboratoire de Recherche et d'EtudeenAménagement et Urbanisme (LREAU), Université des Sciences et de la Technologie (USTHB), Algiers 16000, Algeria
6 Environmental Research Center (CRE), Badji-Mokhtar Annaba University, Annaba 23000, Algeria
7 Institute for Mechanics of Materials, University of Latvia, Jelgavas Street 3, LV-1004 Riga, Latvia
* Correspondence: salma@wus.edu.pk (S.G.); rznkhattak@sbbwu.edu.pk (R.K.)

Citation: Gul, S.; Gul, A.; Gul, H.; Khattak, R.; Ismail, M.; Khan, S.U.; Khan, M.S.; Aouissi, H.A.; Krauklis, A. Removal of Brilliant Green Dye from Water Using *Ficus benghalensis* Tree Leaves as an Efficient Biosorbent. *Materials* **2023**, *16*, 521. https://doi.org/10.3390/ma16020521

Academic Editors: Carlos Manuel Silva, Barbara Gawdzik, Eduarda Pereira and Cláudia B. Lopes

Received: 22 October 2022
Revised: 30 November 2022
Accepted: 28 December 2022
Published: 5 January 2023

Copyright: © 2023 by the authors. Licensee MDPI, Basel, Switzerland. This article is an open access article distributed under the terms and conditions of the Creative Commons Attribution (CC BY) license (https://creativecommons.org/licenses/by/4.0/).

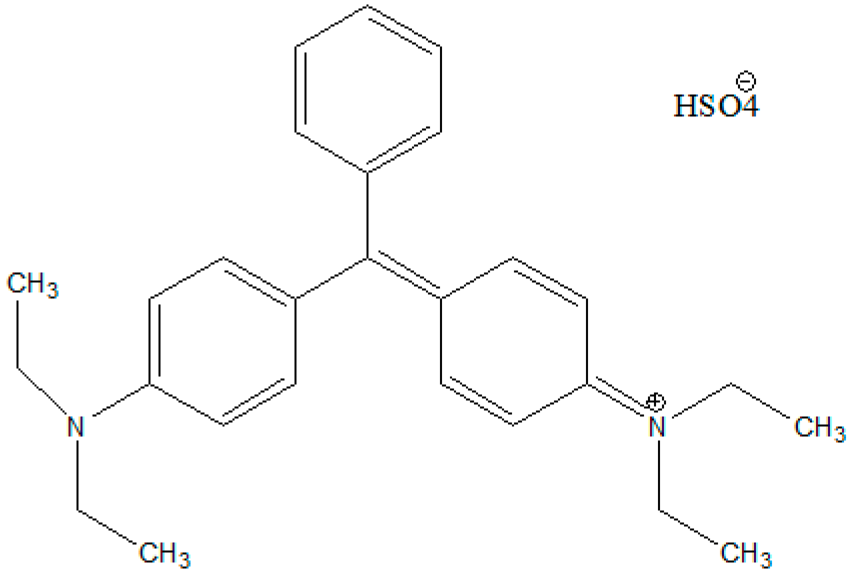

Figure S1. Structural formula of brilliant green dye.
